# Visualization of yellow fever virus infection in mice using a bioluminescent reporter virus

**DOI:** 10.1080/22221751.2021.1967705

**Published:** 2021-09-02

**Authors:** Hao-Long Dong, Hong-Jiang Wang, Zhong-Yu Liu, Qing Ye, Xiao-Ling Qin, Dan Li, Yong-Qiang Deng, Tao Jiang, Xiao-Feng Li, Cheng-Feng Qin

**Affiliations:** a State Key Laboratory of Pathogen and Biosecurity, Institute of Microbiology and Epidemiology, Academy of Military Medical Sciences, Beijing, People’s Republic of China; b Department of Comprehensive Basic Experiment, The Chinese People's Liberation Army Strategic Support Force Characteristic Medical Center, Beijing, People’s Republic of China; c The Center for Infection and Immunity Studies, School of Medicine, Sun Yat-sen University, Guangzhou, People’s Republic of China; d Research Unit of Discovery and Tracing of Natural Focus Diseases, Chinese Academy of Medical Sciences, Beijing, People’s Republic of China; e Department of Medicine and Health, Guangxi Vocational and Technical Institute of industry, Nanning, People’s Republic of China; f Department of Pharmacology, Chinese Academy of Medical Sciences, Beijing, Republic of China

**Keywords:** Reporter virus, *in vivo* imaging, yellow fever virus, mouse model, nano-luciferase

## Abstract

Yellow fever virus (YFV) is a re-emerging flavivirus, which can lead to severe clinical manifestations and high mortality, with no specific antiviral therapies available. The live-attenuated yellow fever vaccine 17D (YF17D) has been widely used for over eighty years. However, the emergence of yellow fever vaccine-associated viscerotropic disease (YFL-AVD) and yellow fever vaccine-associated neurotropic disease (YFL-AND) raised non-negligible concerns. Additionally, the attenuation mechanism of YF17D is still unclear. Thus, the development of convenient models is crucial to understand the mechanisms behind YF17D attenuation and its adverse effects. In this work, we generated a reporter YF17D expressing nano-luciferase (NLuc). *In vitro* and *in vivo* characterization demonstrated that the NLuc-YF17D shared similar biological properties with its parental strain and the NLuc activity can reflect viral infectivity reliably. Combined with *in vivo* bioluminescence imaging, a series of mice models of YF17D infection was established, which will be useful for the evaluation of antiviral medicines and novel vaccine candidates. Especially, we demonstrated that intraperitoneally (i.p.) infection of NLuc-YF17D in type I interferon receptor-deficient mice A129 resulted in outcomes resembling YEL-AVD and YEL-AND, evidenced by viral replication in multiple organs and invasion of the central neuronal system. Finally, *in vitro* and *in vivo* assays based on this reporter virus were established to evaluate the antiviral activities of validated antiviral agents. In conclusion, the bioluminescent reporter virus described herein provides a powerful platform to study YF17D attenuation and vaccine-associated diseases as well as to develop novel countermeasures against YFV.

## Introduction

Yellow fever virus (YFV) is the prototypic member of genus *Flavivirus*, which includes many other vector-borne human pathogens, such as dengue virus (DENV) and Japanese encephalitis virus (JEV). YFV has a single-stranded, positive-sense RNA genome approximately 11 kb in length. Viral polyprotein precursor is translated from a single open reading frame (ORF), which is flanked by two highly structured untranslated regions (UTRs). Host and viral proteases cleave the polyprotein precursor into three structural proteins (C, prM/M and E) and seven non-structural proteins (NS1-NS5).

Human infections of YFV can result in severe yellow fever disease (YF). Mortality rate for severe YF cases is from 20 to 50% [1]. Because of rapid urbanization and global climate change, mosquito-borne flaviviruses kept emerging and re-emerging in the past decade [2–4], posing a persistent threat to public health. Especially, YF, a once rampaging infectious disease in human history [5], is re-emerging in the last few decades. In 2015, a large YF outbreak had occurred in Angola and subsequently spread to the Democratic Republic of Congo, causing 961 confirmed cases and 137 deaths [3]. The YF outbreak in Africa and South America in 2018 caused an estimated 109,000 severe infections and 51,000 deaths [6], and there are still approximately 29,000–60,000 YF-related deaths in Africa and Central and South America annually, according to the WHO [7].

YF is a vaccine-preventable disease, and the live-attenuated YF vaccine 17D (YF17D) has been approved for human use for more than 80 years. A single immunization with YF17D can provide lifetime protection in up to 99% of vaccinees [8]. However, vaccine-associated viscerotropic disease and neurotropic disease have been reported in recent years [9–14]. The mechanism of action behind these serious adverse effects remains largely unknown. Moreover, the molecular mechanisms about YF17D attenuation are not fully understood. On the other hand, albeit several candidate antivirals including adenosine analog NITD008, oligosaccharyl-transferase modulator NGI-1, and sofosbuvir, have shown promising anti-flavivirus activity in cell culture and animal models [15–17], there are still no approved antivirals for YFV infection clinically. These situations issue an urgent call for new platforms which can facilitate the understandings of the attenuation and residential pathogenicity of YF17D as well as the development of antiviral therapies.

Bioluminescent imaging (BLI) technology allows for the noninvasive study of ongoing biological processes in live animals, and has been widely applied in monitoring transgene expression, progression of infection, tumour growth and metastasis, transplantation, and gene therapy. Combined with recombinant viruses expressing specific reporter genes, BLI permits the real-time monitoring of the spatial and temporal progression of viral infection within the same animal. The *in vivo* replication and dissemination dynamics can be easily quantified and visualized by monitoring the bioluminescence magnitude. Nano-luciferase (NLuc), developed in 2012, is the smallest, but the brightest bioluminescent reporter so far [18]. Several engineered reporter viruses carrying the NLuc reporter, including human enterovirus 71, influenza A virus and rabies virus [19–21], have been developed. Yet, there is no research reporting the *in vivo* investigation of NLuc-based reporter flavivirus to our knowledge.

In the present study, we rationally designed and constructed a recombinant YFV reporter virus that efficiently expresses the NLuc based on the infectious clone of YF17D. The resulting reporter virus (named NLuc-YF17D) shared similar characteristics with its parental virus YF17D *in vitro* and *in vivo*, and the magnitude of NLuc activity correlated well with viral infectious titres. Further characterization of NLuc-YF17D in multiple animal models demonstrate the potential applications in novel vaccine and antiviral development.

## Results

### Generation and characterization of the NLuc-YF17D reporter virus

**Figure 1.**
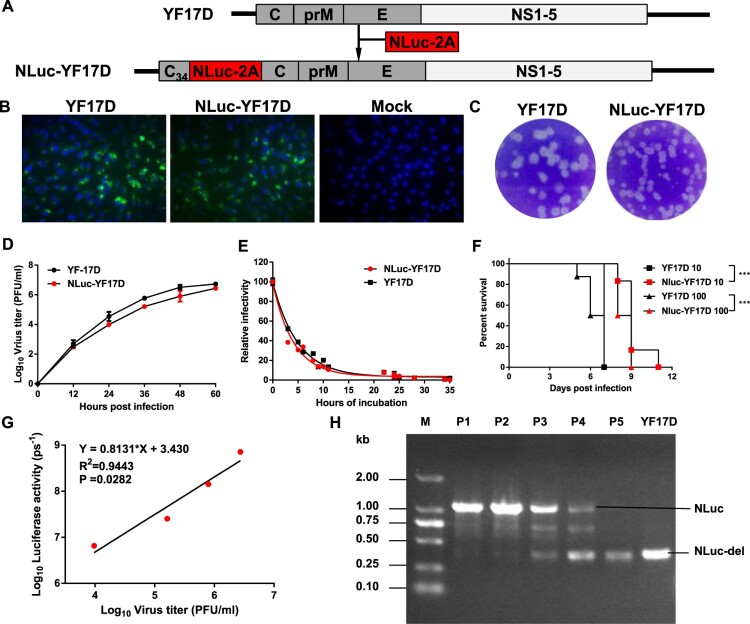
Construction and characterization of NLuc-YF17D. (A) Strategy for the construction of the infectious cDNA clone of NLuc-YF17D. (B) Viral E protein expression in BHK-21 cells infected with the parental YF17D and NLuc-YF17D at an MOI of 1. The IFA was performed at 24 h post-infection. (C) Plaque morphology of YF17D and NLuc-YF17D in BHK-21 cells. (D) Growth curves of the parental YF17D and NLuc-YF17D in Vero cells. Cells were infected with viruses at an MOI of 1, and viral titres in the culture supernatant were determined by plaque assay on BHK-21 cells. (E) Thermal stability assay. Aliquots of 1000 PFU of the corresponding viruses in 1 ml DMEM were incubated at 40°C for the indicated period of time and titred by plaque assay on BHK-21 cells. (F) One-day-old BALB/c suckling mice (*n* = 6–8 per group) was i.c. infected with the indicated dose of viruses and monitored daily for 11 days to assess morbidity and mortality. Kaplan–Meier survival curves were analyzed by Log-rank (Mantel–Cox) test using standard GraphPad Prism software 6.0; ***extremely significant (*p* < 0.001). (G) Viral titre was plotted against luciferase activity in the culture supernatant. Vero cells were infected with NLuc-YF17D at an MOI of 1 and culture supernatant was collected at indicated time points and stored at −80°C. Bioluminescent signals and viral titre in the collected culture supernatant were determined by luciferase assay and plaque assay, respectively. (H) Genetic stability of NLuc-YF17D. RT-PCR was performed to amplify the region containing the NLuc gene for the serially passaged viruses. The size of the region containing the NLuc gene is 997 bp, and it gets 415 bp after the loss of NLuc gene.

To generate a replication-competent YFV reporter virus, we firstly engineered the NLuc gene followed by FMDV-2A auto-proteolytic sequence, into the full-length infectious cDNA clone of YF17D. The NLuc-2A sequence was inserted downstream of the sequence encoding the first 34 residues of C protein (C_34_) since *cis*-acting RNA elements essential for viral replication [22], located within the coding sequence of C_34_. The NLuc-2A sequence was followed by an intact viral polyprotein ORF (Figure 1(A)). The 5’ cyclization sequence in the full-length C-coding region was disrupted by introducing synonymous mutations in order to abolish their possible interference with the correct mode of genome cyclization [23]. After *in vitro* transcription and transfection, the resulted recombinant reporter virus, named NLuc-YF17D, was successfully rescued and prepared in BHK-21 cells.

A panel of assays was then performed to characterize the NLuc-YF17D reporter virus in comparison with its parental virus YF17D. Firstly, by indirect immunofluorescence assay, we showed that NLuc-YF17D and YF17D-infected BHK-21 cells were both positive for YFV E protein (Figure 1(B)). Next, we found that NLuc-YF17D formed slightly small plaques in BHK-21 cells compared with YF17D (Figure 1(C)), and they had similar growth kinetics in Vero cells (Figure 1(D)). Moreover, thermostability was also compared between NLuc-YF17D and the parental YF17D using a recently described method [24]. The loss of infectivity assay at 40°C showed that NLuc-YF17D and YF17D shared a similar half-life time, thus the genetic engineering of YF17D did not change its thermostability apparently (Figure 1(E)).

To characterize the *in vivo* phenotype of NLuc-YF17D, the suckling mice model, which is widely used for virus neurovirulence test, was utilized [25–29]. We infected one-day-old BALB/c suckling mice (*n* = 6–8 per group) through intracranial injection of 10 or 100 PFU (plaque forming unit) of NLuc-YF17D and its parental virus, YF17D. The infected subjects were monitored daily for 11 days to assess morbidity and mortality. All the suckling mice succumbed to infection at the given doses of the two viruses in 11 days, with median survival values of 9, 7, 8.5, 6.5 days for NLuc-YF17D 10, YF17D 10, NLuc-YF17D 100, YF17D 100 groups respectively (Figure 1(F)). The log-rank (Mantel–Cox) analysis shows that there were statistically significant differences between the survival time of the NLuc-YF17D and YF17D group at the same infection dose (*p* < 0.001), but the infection of the NLuc-YF17D also caused a 100% lethality in one-day-old suckling mouse model, which is in consistent with its parental YF17D strain. Together, the above results demonstrated that the biological characteristics of NLuc-YF17D agreed well with its parental strain.

To confirm that bioluminescent signals can be a read-out of viral titres *in vitro,* we plotted viral titres in the supernatant against bioluminescent signals and linear regression analysis showed that they are positively correlated with each other (Figure 1(G)). At last, serially passaging of NLuc-YF17D in BHK-21 cells showed that NLuc gene was completely lost after five passages (Figure 1(H)), which is in agreement with previous studies on reporter viruses including JEV [30] and DENV [31]. Thus, only the first and second passage viral stocks of NLuc-YF17D were used in subsequent experiments for consistency.

### Monitoring NLuc-YF17D infection in suckling mice by BLI.

**Figure 2.**
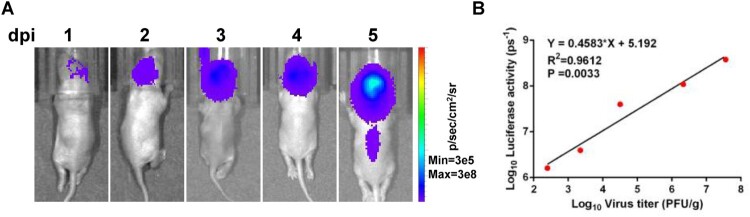
Viral replication in BABL/c suckling mice. (A–B) In vivo correlation between bioluminescent signals and viral replication. (A) Bioluminescent images of infected animals at indicated time points. (B) Quantified viral titres in the excised brains were plotted against quantified bioluminescent signals in head areas of animals in (A). One-day-old BALB/c suckling mice was infected through intracranial injection with NLuc-YF17D (1000 PFU) and subjected to imaging at indicated time points. The brains were harvested immediately after performing BLI and stored at −80°C. Bioluminescent signals were quantified using Living Image software 3.0. Viral titres in the excised brains were quantified by plaque assay.

The suckling mouse model was employed again to observe whether the NLuc-17D can be used for *in vivo* BLI. One-day-old BALB/c suckling mice were infected through intracranial injection with NLuc-YF17D (10^3^ PFU) and subjected to imaging from day 1 to day 5 post-infection (Figure 2(A)). The brains were harvested immediately after performing BLI. Viral titres in the excised brains were quantified by plaque assay. Quantified viral titres in the excised brains were plotted against quantified bioluminescent signals in head areas of the animals (Figure 2(B)). Linear regression analysis showed that NLuc activity can be used as a read-out of viral replication. Taken together, these data demonstrated that NLuc-YF17D is qualified as a reporter virus for application.

**Figure 3.**
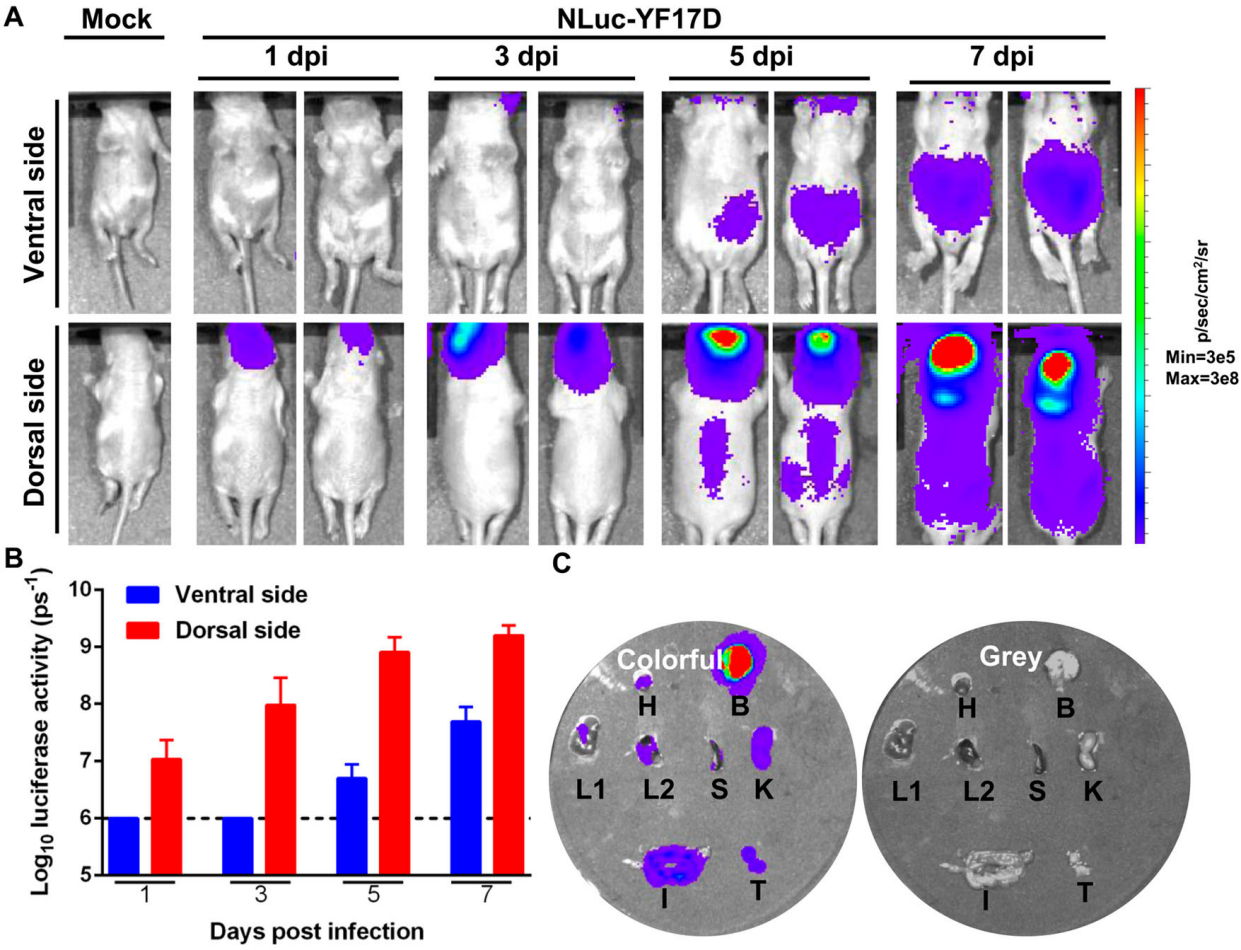
NLuc-YF17D causes a systemic infection in suckling BALB/c mice. Bioluminescence imaging of suckling BALB/c mice reveals systemic infection with NLuc-YF17D. One-day-old suckling BALB/c mice (*n* = 3) was infected through intracranial injection with NLuc-YF17D (1000 PFU). BLI was performed at indicated time points in whole animals (A) and bioluminescent signals were quantified using Living Image software 3.0 (B). (C) Animals were euthanized at 7 days post-infection immediately after BLI and organs including heart (H), brain (B), liver (L1), lung (L2), spleen (S), kidney (K), intestine (I), testicles (T) were harvested for ex vivo BLI. Images of the isolated organs with or without bioluminescence were shown on the left and the right, respectively.

To further explore the viral dynamics and dissemination of NLuc-YF17D in suckling mice, a group of one-day-old suckling BALB/c mice was infected with 10^3^ PFU of NLuc-YF17D through intracranial injection and monitored for disease onset. On days 1, 3, 5, 7 post-infection, BLI was performed to observe *in vivo* virus propagation. Bioluminescent signals were detected at the injection site at 1 d post-infection (dpi) and at abdomen areas at 5 days post-infection (Figure 3(A)), with the strongest bioluminescent signals in the head areas over the course of infection (Figure 3(B)). The moribund mice were euthanized and dissected immediately after the BLI, and the isolated organs were subjected to imaging. Multiple organs, including the brain, heart, liver, lung, spleen, kidney, intestine and testicle, were positive for NLuc. These results suggested that a systemic infection of YF17D can be established through intracerebral inoculation of suckling mice (Figure 3(C)).

### Establishment of a BLI-based animal model of YEL-AVD and YEL-AND

**Figure 4.**
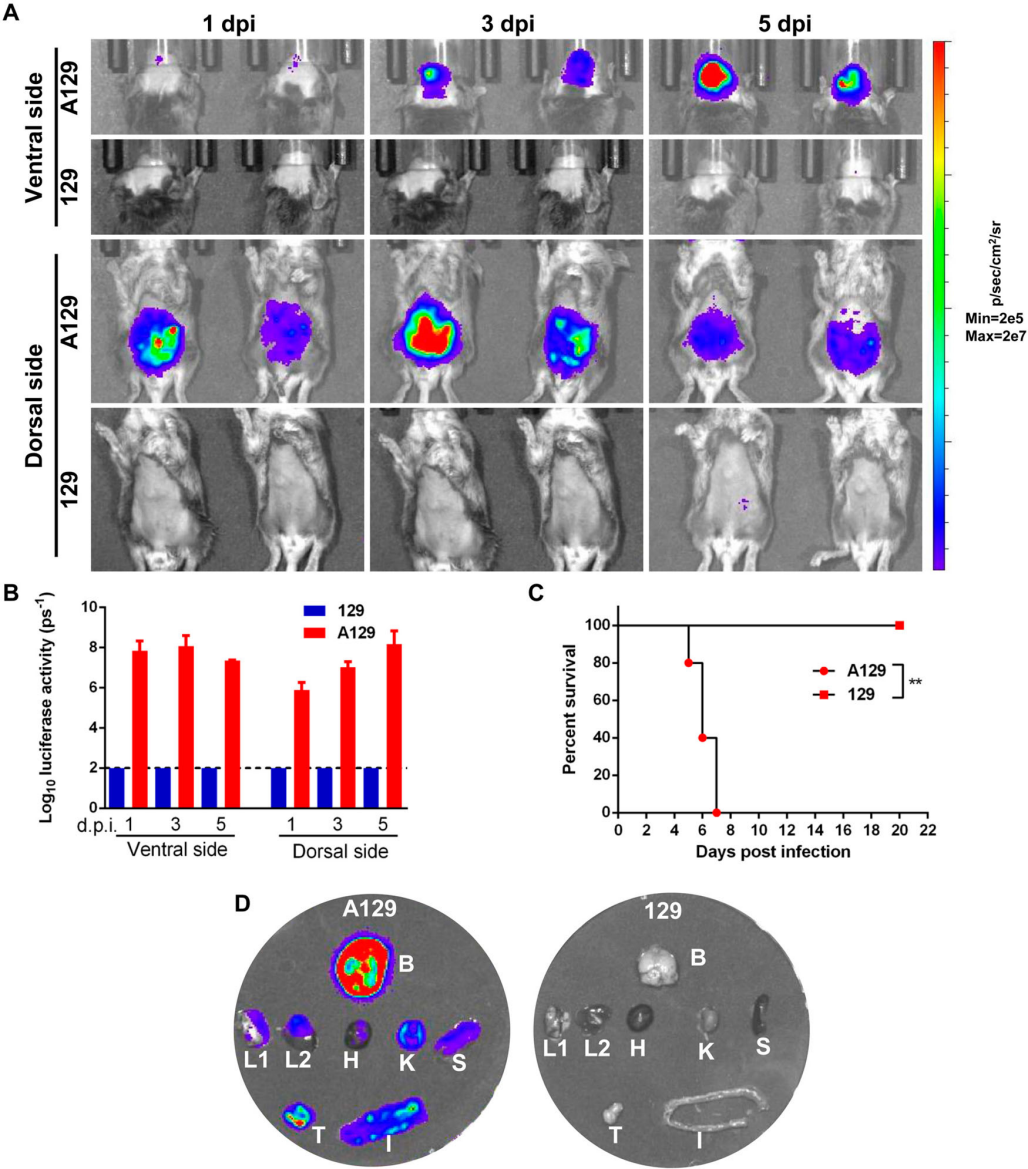
NLuc-YF17D causes viscerotropic and neurotropic diseases in two-week-old A129 mice. Two-week-old A129 or 129 mice were i.p. inoculated with 1000 PFU of NLuc-YF17D. (A) Bioluminescence imaging of A129 and 129 (*n* = 3) mice were performed at indicated time points. (B) Bioluminescent signals were quantified using Living Image software 3.0. (C) Survival analysis of two-week-old mice infected with NLuc-YF17D. Kaplan–Meier survival curves were analyzed by log-rank test using standard GraphPad Prism software 6.0. **, *P* < 0.01. (D) Representative subjects of each group were euthanized and dissected at 5 days post-infection. The organs including heart (H), brain (B), liver (L1), lung (L2), spleen (S), kidney (K), intestine (I), testicles (T) were harvested for ex vivo BLI.

There were only a few animal models that can be used for the investigation of YEL-AVD and YEL-AND. In order to establish a convenient, BLI-based model for these adverse effects, a group of two-week-old type I interferon (IFN) receptor-deficient mice (A129) were i.p. infected with 10^3^ PFU of NLuc-YF17D, immunocompetent 129 mice of the same age were infected under the same conditions as controls. Serial imaging revealed that robust bioluminescent signals were detected in the abdomen in A129 mice as early as 1 dpi, suggesting that the two-week-old A129 mice were highly susceptible to YF17D (Figure 4(A)). At 3 dpi, replication of NLuc-YF17D in mouse brains was observed by BLI imaging (Figure 4(A)). Upon the detection of NLuc activity in the brain, neurotropic manifestations including ruffled fur, lethargy, hind limbs paralysis and hunched posture were observed in the A129 mice. Bioluminescent signals in the brain continuously increased over the course of infection in A129 and peaked at 5 dpi when infected subjects began to succumb to infection (Figure 4(B,C)). Thus, YF17D retained sufficient neuroinvasiveness to break the blood–brain barrier of the two-week-old A129 mice. By contrast, no bioluminescent signal was detected in the control 129 mouse group, which remained healthy throughout the experiment (Figure 4(B,C)). These results are consistent with previous findings that type I IFN signaling control of flavivirus infection [32].

To confirm the infection of individual organs by NLuc-YF17D, at 5 dpi, a morbid A129 and a control 129 mouse were euthanized and dissected immediately after imaging and isolated organs were imaged. Bioluminescent signals were detected in multiple A129 mouse organs including liver, lung, heart, spleen, kidney, intestine, testis and brain. Notably, the strongest bioluminescent signal was detected in the brain (Figure 4(D)). Taken together, the above results demonstrated that two-week-old A129 mouse is highly susceptible to the attenuated YF17D strain, and the organic tropism of the resulted infection resembles the YEL-AVD and YEL-AND in humans. Thus, our results suggested that the BLI-based animal model described above will be useful for the investigation of the pathogenesis of these serious side-effects.

### The outcome of YF17D infection depends on the inoculation route in A129 mice.

**Figure 5.**
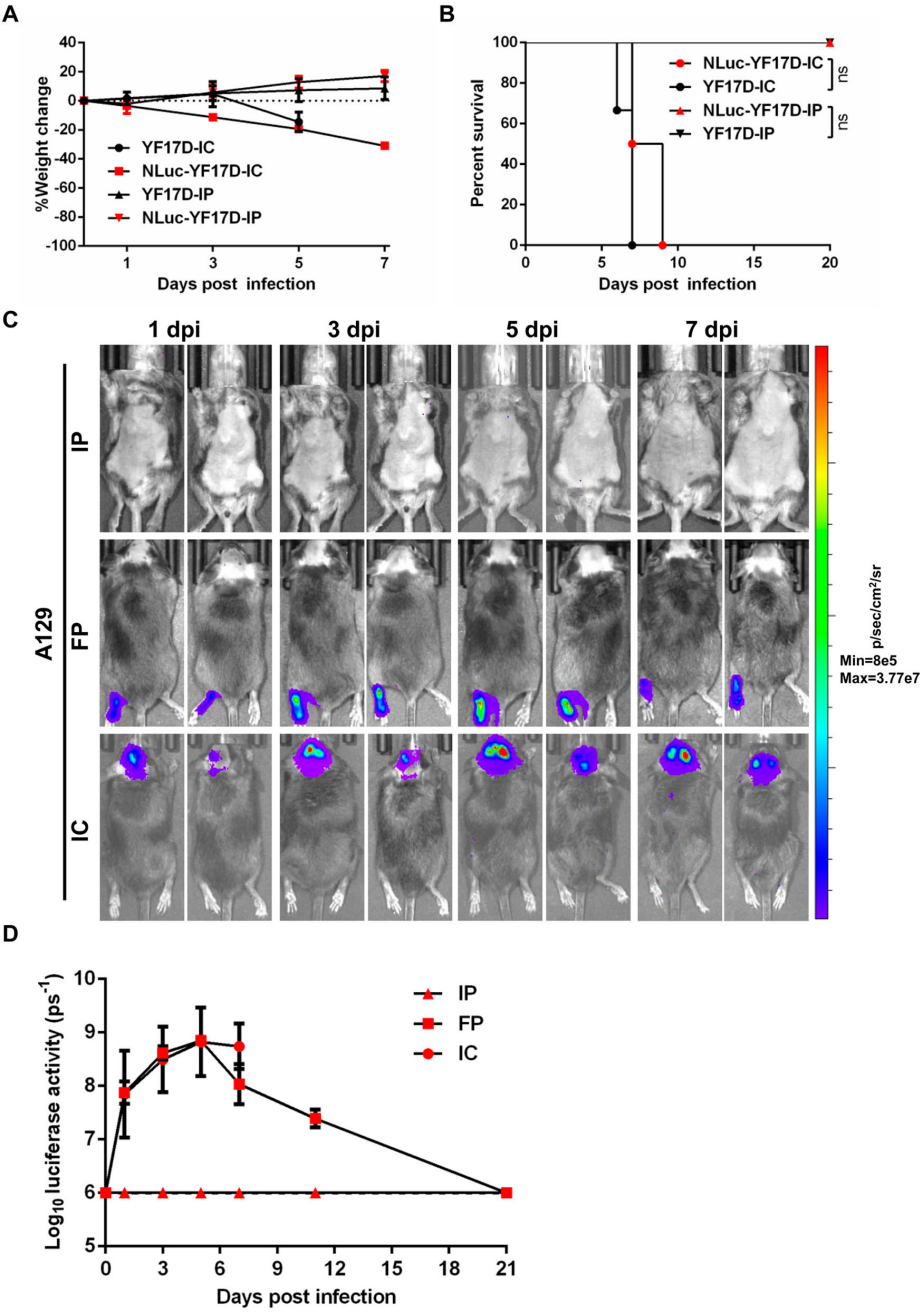
The outcome of NLuc-YF17D infection in four-week-old A129 mice is route-dependent. (A–B) Four-week-old A129 mice were i.c. or i.p. infected with 1000 PFU NLuc-YF17D or its parental virus (*n* = 2–3 per group). (A) Weight change was recorded. (B) Kaplan–Meier survival curves were analyzed by Log-rank (Mantel–Cox) test using standard GraphPad Prism software 6.0; ns: not significant (*p* > 0.05). (C–D) Four-week-old A129 (*n* = 2) mice was infected through intraperitoneal, footpad or intracranial injection with 1000 PFU NLuc-YF17D. (C) BLI was performed at the indicated time points. (D) Bioluminescent signals of the mice were quantified using Living Image software 3.0.

A previous study [33] reported that the 17D-204 vaccine strain is avirulent in three-to-four-week-old A129 mice through footpad administration, whereas the parental Asibi strain is highly virulent. However, Erickson et al found that 17D strain was still pathogenic when i.p. inoculated in a C57BL/6 based type I IFN receptor-deficient mouse model, suggesting that type I IFN plays an important role in attenuation of the 17D vaccine [34]*.* Thus, we firstly evaluated the pathogenicity of YF17D and NLuc-YF17D in four-week-old A129 mice. It was shown that i.c. injection of parental YF17D or recombinant NLuc-YF17D (10^3^ PFU) led to neurological disease and weight loss, which were not seen in i.p. inoculated mice (Figure 5(A)), consistent with previous report [35]. Unsurprisingly, both YF17D and NLuc-YF17D infection via intracranial injection led to robust virus replication in the brains, followed by decease of the infected mice (Figure 5(B)). Thus, the route of inoculation is a determinator of infection of NLuc-YF17D or YF17D in A129 mouse.

To further confirm the role of inoculation route in the infection outcomes, we then performed BLI of NLuc-YF17D infection using this model with a panel of different inoculation routes. Groups of four-week-old A129 mice were injected with NLuc-YF17D via intraperitoneal (IP), footpad (FP), or intracranial (IC) route and serially imaged. Firstly, we found that the four-week-old A129 mice were resistant to NLuc-YF17D infection via intraperitoneal injection, as no bioluminescent signal was detected throughout the experiments, and all subjects remained healthy (Figure 5(C,D)). Secondly, A129 mice inoculated with NLuc-YF17D via footpad injection also showed no clinical manifestations, agreeing with the previous report [33]. Interestingly, BLI indicated that NLuc-YF17D replicated limitedly at the injection site in FP group of A129 mice before its clearance (Figure 5(C,D)). This finding also highlighted the sensitivity of NLuc-YF17D-based BLI, as this kind of limited replication is hardly detectable through conventional methods. Thus, using BLI, we confirmed that the route of inoculation is a critical factor that influenced the clinical outcome of NLuc-YF17D infection in A129 mouse. In addition, animal age is also an important factor of the infection outcomes, since NLuc-YF17D i.p. infects two-week A129 (Figure 4(A)) but not four-week-old ones (Figure 5(C)). Importantly, these results confirmed that the pathogenicity of YF17D and NLuc-YF17D is similar in the four-week-old A129 mouse model. The different mouse infection models described herein can be used to meet the specialized needs of different researches.

### *In vitro* and *in vivo* evaluation of antivirals using the NLuc-YF17D.

**Figure 6.**
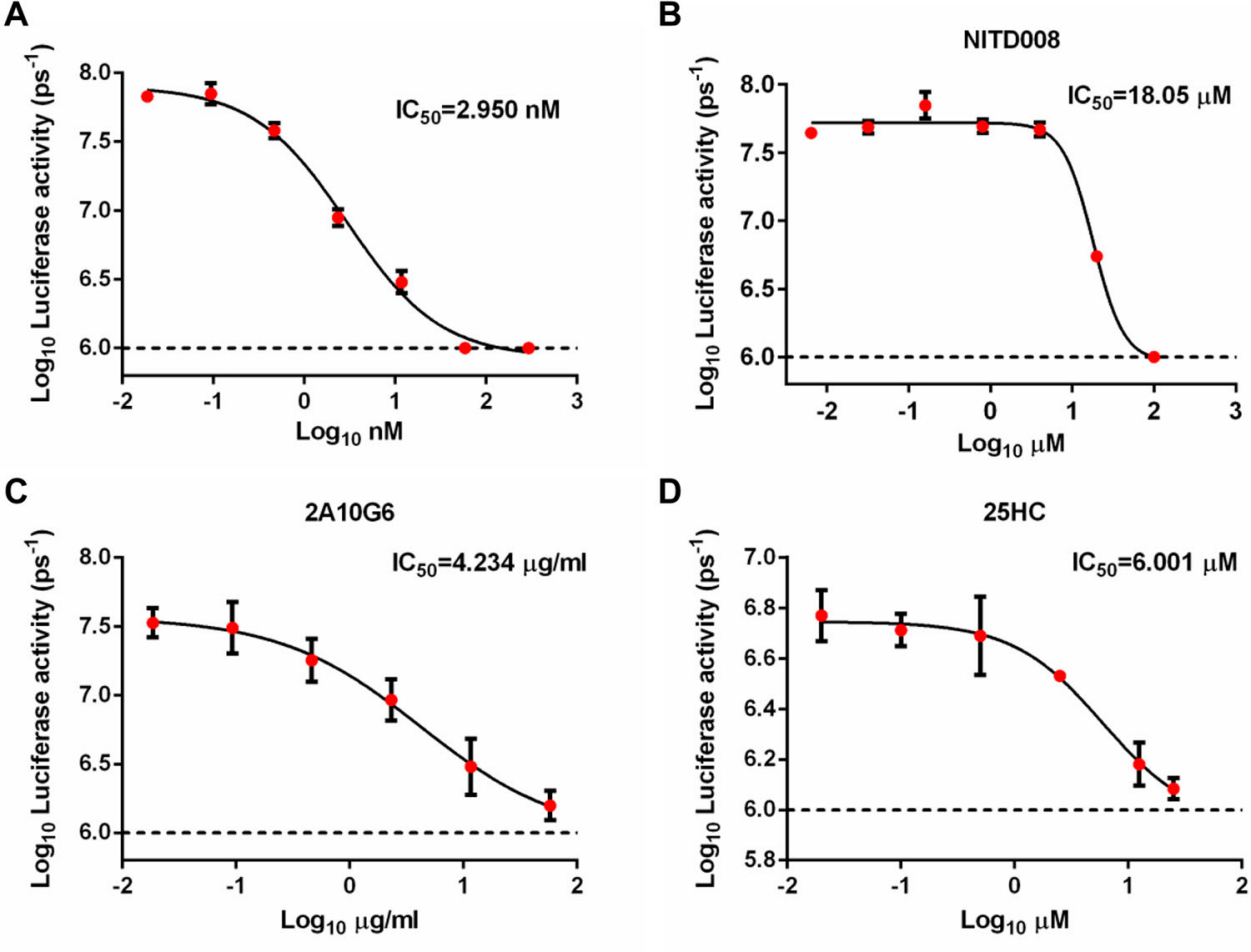
In vitro evaluation for antivirals using NLuc-YF17D. Temoporfin (A), NITD008 (B), 2A10G6 (C) and 25HC (D) inhibited the replication of NLuc-YF17D in vitro. Luciferase assay of inhibition of NLuc-YF17D by different concentrations of antivirals was quantified by ROI analysis using Living Image 3.0. The 50% inhibitory concentration (IC50) values were calculated by fitting the Log (dose)-response curves from bioluminescence reduction assay.

Given the fact that no approved anti-YFV medicine is available, one of the most important applications of reporter virus would be high-throughput screening for antiviral compounds. We next tested a few reported anti-flavivirus compounds with our reporter virus system. These compounds include temoporfin [36], NITD008 [37], the mAb 2A10G6 [38], and 25-hydroxycholesterol [39]. Dose-dependent antiviral effect was observed for all tested compounds by the reporter virus system, with IC_50_ for Temoporfin 2.950 nM (Figure 6(A)), NITD008 18.05 µM (Figure 6(B)), 2A10G6 4.234 µg/ml (Figure 6(C)), 25HC 6.001 µM (Figure 6(D)).

**Figure 7.**
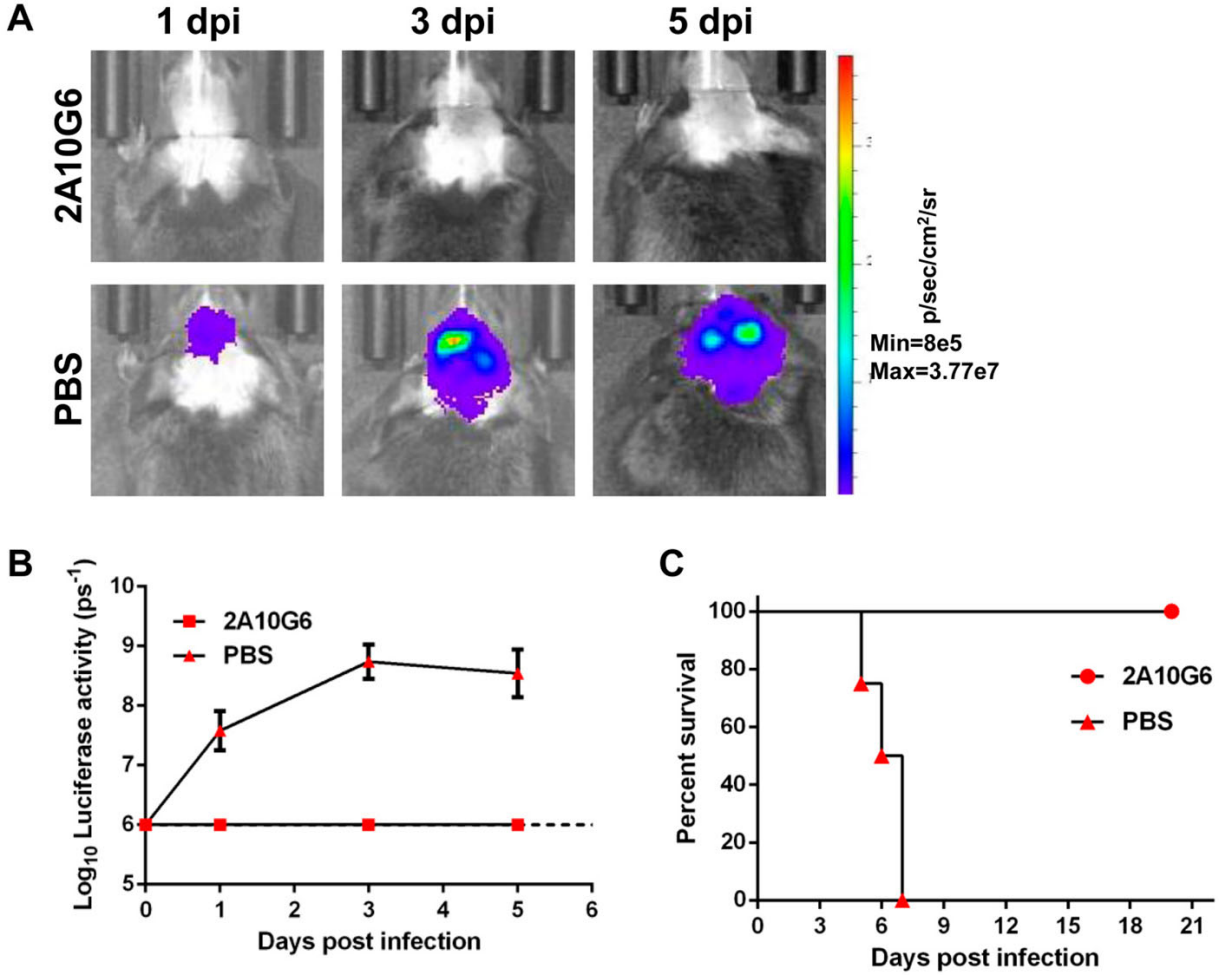
Inhibition of in vivo replication of NLuc-YF17D by the mAb 2A10G6. Four-week-old A129 mice (*n* = 2–4) were i.c. inoculated with a mixture of 100?g of 2A10G6 and 1000 PFU of NLuc-YF17D. The control group was inoculated with a mixture of the same volume of PBS and 1000 PFU of NLuc-YF17D. BLI was performed at indicated time points and mortality was monitored daily for 21 days. (A) A representative image series for the 2A10G6 group and the control PBS group were presented. (B) Bioluminescent signals were quantified for the two groups. (C) Survival curves of the infected mice were drawn.

We next chose 2A10G6 to test its *in vivo* inhibitory effect against NLuc-YF17D. To this aim, a group of four-week-old A129 mice were intracranially (i.c) injected with a mixture of 2A10G6 and NLuc-YF17D. For the control group, Nlu-YF17D was mixed with an equal volume of PBS. BLI was performed at 1, 3 and 5 dpi. Robust bioluminescent signals were detected in the subjects of the control group, and the signal increased as the inoculation period extended, suggesting the establishment of infection. On the other hand, no bioluminescent signal was detected in the 2A10G6 group throughout the experiment, suggesting that the virus had been neutralized by the mAb. In agreement with the above results, the control group developed a severe neurotropic disease and succumbed to infection at 6 or 7 dpi while the 2A10G6 group showed no sign of diseases and all survived (Figure 7(A–C)). Additionally, we have also treated the infected animals with 2A10G6 at −1 and 2 dpi, and BLI results showed that administration of 2A10G6 separately also inhibited the replication of NLuc-YF17D in A129 mice (Fig. S1). These results not only validated the potential applications of NLuc-YF17D in antiviral screening but also highlighted that our *in vivo* imaging model will facilitate the efficacy evaluation of antiviral candidates.

## Discussion

As the first recognized human viral pathogen, YFV has a long history of epidemics. Reporter virus system has been developed for many viruses, such as JEV [30], DENV [31] and influenza virus [20,40]. To the best of our knowledge, this study is the first to apply the combination of reporter virus and BLI into the research of YFV. In this work, firstly the NLuc-YF17D reporter virus was generated by reverse genetics approaches. After a series of virological characterization, the NLuc-YF17D was used to infect mice of different age and immune status via different infection routes, and the viral replication dynamics and outcomes of the infection were investigated.

Firstly, we established a suckling mouse model of NLuc-YF17D. Through BLI, we showed that NLuc-YF17D replicated robustly in the brain after inoculation, and was able to disseminate to multiple visceral organs. The exact mechanism about how NLuc-YF17D transports out from the brain may require further investigations. Next, by monitoring the viral dynamics of NLuc-YF17D infection in two-week-old A129 mice infected by intraperitoneal route, we recorded the infection course that NLuc-YF17D overcame brain–blood barrier and spread to visceral organs resulting in a pan-systemic infection. Thus, this infection model will be valuable for the investigation of YEL-AVD and YEL-AND. The high sensitivity of NLuc-YF17D also enabled us to reveal that the attenuation mechanism of YF17D is associated with type I IFN signaling. Additionally, NLuc-YF17D allowed us to visualize its replication in four-week-old A129 mice and revealed its route-dependent and animal-age-dependent infection results. These models will also facilitate the assessment of antiviral medicines and are helpful for next-generation vaccine development. Finally, the potential applications of NLuc-YF17D in the evaluation of anti-YFV activity were demonstrated by testing a series of previously reported antivirals.

Interferon receptor deficient mouse models have been established using wild-type Asibi virus or YF17D strain [33,34]. Although this animal model mimicked viscerotropic disease caused by YFV in human, details about the replication dynamics were missed due to inherent limitations of conventional mouse model. Using BLI, our mouse models of NLuc-YF17D infection allowed us to monitor the course of infection in the same animals. Also, high sensitivity of this system enabled us to visualize low level of viral replication *in vivo*, as shown in A129 mouse models via the footpad inoculation route. YF17D-asscociated adverse events have been reported for decades. However, the corresponding molecular mechanism still remains unknown. With the animal models reported herein, NLuc-YF17D is in a good position to represent its parental YF17D in understanding the pathogenesis behind YF17D-associated adverse events.

However, NLuc-YF17D is not genetically stable in that NLuc gene was lost after 5 passages in Vero cells. Plaque-purification is a possible way of overcoming genetic instability of reporter virus, as we did with JEV [30]. Although insertion of NLuc gene leads to attenuation of the parental YF17D (Figure 1(F)), NLuc-YF17D mimics most virological characteristics of its parental virus (Figure 1(C–F), 5(C–D)). Furthermore, NLuc-YF17D-based animal models have advantages beyond their conventional counterparts. NLuc is much smaller and brighter than firefly luciferase and renilla luciferase. However, unlike firefly luciferase whose substrate is able to distribute throughout the whole body, NLuc’s substrate has limited ability to diffuse in animal body. Moreover, injection of substrate is needed for NLuc activity to be detected each time. Frequent injection of substrate surely inflicts injury upon animals. Further work needs to be done to improve NLuc-YF17D to widen its application. Gaussia princeps luciferase (GLuc) has the molecular weight as NLuc, but it has a lower signal-to-noise ratio and a much shorter signal half-life, limiting its application *in vivo* [41]. In our experiments, we noticed that it was better to detect NLuc activity in the brain from the dorsal side (Figure 3(A)), visceral organs from the ventral side (Figure 4(A)). A better signal-to-noise ratio was achieved when fur on the region of interest was removed before imaging. The reason behind this is that the bioluminescent signal is largely blocked in animal bodies.

## Materials and methods

### DNA manipulation

The infectious clone pACNR-FLYF17Dx of YF17D [22] was kindly provided by Professor Charles M. Rice (the Rockefeller University). The NanoLuc gene was inserted into the 17D cDNA by overlapping PCR. Briefly, the DNA fragment, which contains the SP6 promoter, viral 5′UTR and the first 102-nt of C gene was fused with the NanoLuc gene followed by the coding sequence of FMDV 2A peptide by first-round overlapping PCR. Then the PCR product was further fused upstream of a DNA fragment containing the 1–1574 nt of viral polyprotein ORF by second-round overlapping PCR. The sequence required for genome cyclization in the full-length C ORF was also disrupted by introducing synonymous mutations, in order to abolish their possible interference with normal viral RNA replication [22,42]. The final PCR product was sub-cloned into the pACNR/FLYF17Dx plasmid by Not I/Nsi I digestion. The resultant plasmid was designated as pACNR- NLuc-YF17D. The primers used in this study was listed in Table S1.

### Virus preparation

BHK-21 cells (ATCC CCL-10) were grown in Dulbecco’s modified Eagle medium (DMEM) with 25 mM HEPES (Gibco), 10% fetal bovine serum (FBS) (Gibco) and 100 U/ml penicillin–streptomycin (P/S, Gibco) at 37°C in a 5% CO_2_ atmosphere. The pACNR-YF17D-NanoLuc and pACNR/FLYF17Dx plasmid was linearized by Xho I digestion and used as the templates for SP6-directed *in vitro* transcription. The transcripts were purified by using an RNeasy Mini Kit, quantified spectrophotometrically and the integrity was checked by TAE/agarose gel electrophoresis. Then the transcripts were transfected into BHK-21 cells by using the GenePulser Xcell electroporation system (Bio-rad). After 48 h, culture supernatants were collected, clarified by centrifugation and stored at −80°C as single-use aliquots. Infectious virus titres were determined by plaque assay on BHK-21 cells, expressed as plaque-forming units (PFU)/ml.

### Immunofluorescence assay (IFA)

Monolayer of BHK-21 cell, seeded on a Chamber Slide (Thermo fisher scientific), was infected with the parental YF17D or NLuc-YF17D at an MOI of 1. At 24 h post-infection, the cells were fixed with acetone. The fixed cells were washed three times with PBS and then incubated with mouse Anti-Flavivirus E-glycoprotein monoclonal antibody (1:200 dilution with PBS, Abcam) for 1 h. After washing with PBS three times, the cells were incubated with goat anti-mouse IgG conjugated with Alexa Fluor 488 (ZSBIO, China) at room temperature for 1 h. After three times of washing with PBS, the cells were incubated with DAPI (ZSBIO, China) at room temperature for 5 min. The slide was mounted with water and examined under a fluorescence microscope (Olympus).

### Thermostability assay

Aliquots of 10^3^ PFU of NLuc-YF17D or YF17D in DMEM supplemented with 10% FBS and 100 U/ml P/S, were incubated at 40°C, and sampled periodically for the next 35 h. At each time point, aliquots were collected and stored at −80°C. All frozen samples were thawed simultaneously and used to infect BHK-21 cells in duplicate to assess infectivity by plaque assay. Infection was normalized to the original level (without incubation at 40°C) and fitted with a one-phase exponential decay curve using the GraphPad Prism 6.0 software to estimate the infectious half-life.

### Luciferase assay

BHK-21 cells were infected with NLuc-YF17D at indicated MOIs and culture supernatants were collected at indicated time points and stored at −80°C for subsequent luciferase assay or plaque assay. Luciferase activity was measured in a 96-well plate by mixing 10 μl supernatant with 50 μl substrate (Promega). Luciferase assay was performed using IVIS Spectrum instrument (Perkin Elmer) with 3 s exposure time, 4 binning and 1 Fstop. Each well in the plate was analyzed as a region of interest (ROI) and bioluminescent signals were quantified using Living Imaging 3.0.

### BLI

BLI in mice was performed using IVIS Spectrum instrument (Perkin Elmer) with 3 s exposure time, 4 binning and 1 Fstop. Animals were under anesthesia induced and maintained with isoflurane throughout the imaging procedure. 20 µl Nano-Glo luciferase assay substrate (1:50 dilution with PBS, Promega) was administered via left footpad (FP), intraperitoneally (IP) or intracranially (IC) to each animal 3 min before the imaging. To precisely quantify the amount of light emitted from mice, regions of interest (ROIs) were manually defined around head, abdomen or left leg areas.

### Animal experiments

All animal experiments were performed in accordance with the guidelines of the Chinese Regulations of Laboratory Animals (Ministry of Science and Technology of People's Republic of China) and Laboratory Animal-Requirements of Environment and Housing Facilities (National Laboratory Animal Standardization Technical Committee). The experimental protocols were approved by the Animal Experiment Committee of Beijing Institute of Microbiology and Epidemiology, Beijing, China. Mouse strains used in this study included one-day-old BALB/c suckling mice, two-week-old, three-week-old, four-week-old wild-type 129/Sv/Ev mice (Beijing Vital River Co. Ltd, China) and 129/Sv/Ev mice deficient in type I IFN receptors (A129) which were kindly provided by Prof. Qi-Bin Leng (Shanghai Institute for Pasteur, CAS, China). All efforts were made in animal experiments to minimize pain and suffering.

For correlation studies, One-day-old BALB/c mice were i.c. inoculated with 10^3^ PFU of NLuc-YF17D diluted in 20 µl DMEM. The infected animals were imaged at 1, 2, 3 or 5 dpi and bioluminescent signals in head areas were quantified using Living Image 3.0. Immediately after imaging, mice were euthanized and brains were harvested and stored at −80°C for subsequent plaque assay. Quantification of infectious virus in the brains by plaque assay was plotted against bioluminescent signals.

For neurovirulence study, one-day-old BALB/c mice (*n* = 6–8 per group) were infected through intracranial injection with YF17D or NLuc-YF17D (10^2^ or 10^3^ PFU) diluted in 20 µl DMEM. Infected animals were monitored daily for morbidity and mortality.

For the tissue distribution study, a group of one-day-old BALB/c mice (*n* = 3) was infected through intracranial injection with 10^3^ PFU NLuc-YF17D diluted in 20 µl DMEM. Infected animals were imaged at 0, 1, 3, 5 and 7 dpi. At 7 dpi, a mouse was euthanized immediately after imaging. Then the brains, liver, spleen, kidney, intestine, testis, heart and lung were collected for *ex vitro* BLI imaging.

For study on effects of type I, IFN and infection route on virulence of NLuc-YF17D, groups of four-week-old A129 or 129 mice (*n* = 2) were infected via left footpad, intraperitoneal or intracranial injection with 10^3^ PFU of NLuc-YF17D. Infected animals were imaged at indicated time points and were monitored for morbidity and mortality.

For *in vivo* inhibition of NLuc-YF17D by 2A10G6 experiment. A129 mice were i.c. inoculated with a mixture of 10^3^ PFU of NLuc-YF17D and 100 μg 2A10G6 or PBS. Additionally, 2A10G6 was separately injected at −1 and 2 dpi for another a group of A129.

### Antiviral assay

BHK-21 cells were seeded into 12-well plates at a density of 1 × 10^5^ cells/well. After 24 h incubation, the cells were infected with NLuc-YF17D at an MOI of 0.1 and incubated with various concentrations of NITD008 or Temoporfin (0–0.294 μM). For 25HC, cells were pre-incubated with 25HC (0–25 μM) 8 h before infection with NLuc-YF17D. Culture supernatants were collected at 48 h post-infection when cytopathogenic effect (CPE) was observed in control cells. The luciferase activity was measured by luciferase assay in triplicate. The IC_50_ was calculated using GraphPad Prism 6.0 software.

## Supplementary Material

Clean_copy_of_table_s1.docx
