# Supplementary material for: Visualization of yellow fever virus infection in mice using a bioluminescent reporter virus
Source: Emerg Microbes Infect. 2021 Sep 2;10(1):1739–50. doi: 10.1080/22221751.2021.1967705 (PMC8425728; doi:10.1080/22221751.2021.1967705)
Supplement: Clean_copy_of_table_s1.docx [file TEMI_A_1967705_SM8162.docx]

**Table S1. Primers used for the generation of the fused Not I-17D-Nluc-Nsi I fragment**

| **Primers** | **Sequence (5′-3′)** | **Purpose** |
| --- | --- | --- |
| **P-NotI-F** | **Ccgaaaagtgccacctgac** | **Preparation of the fragment 1 of the final product** |
| **P-YFC-Nluc-B** | **ccatTCCACTTCCGCCAATTTGTTTTGTTTTTTGTTTTATTTTGTTTGAC** |  |
| **P-YFC-Nluc-F** | **ATTGGCGGAAGTGGAatggtcttcacactcgaagatt** | **Preparation of the fragment 2 of the final product** |
| **P-Nluc-2A-B** | **acagctgTCCAGATCCTgcCagaatgcgttcgcac** |  |
| **P-Nluc-2A-F** | **gcAGGATCTGGAcagctgttgaattttgaccttct** | **Preparation of the fragment 3 of the final product*** |
| **P-NsiI-B** | **GCGGCATGCGGAGGTTCAAAT** |  |
| **P-NotI-F** | **Ccgaaaagtgccacctgac** | **Fusion of the fragment 1 and fragment 2** |
| **P-Nluc-2A-B** | **acagctgTCCAGATCCTgcCagaatgcgttcgcac** |  |
| **P-NotI-F** | **Ccgaaaagtgccacctgac** | **Fusion of the fragment 1+2 and fragment 3** |
| **P-NsiI-B** | **GCGGCATGCGGAGGTTCAAAT** |  |
| **P-YF17D-F1** | **CGTTCGTTGAGCGATTAGCAGAG** | **Amplification of Nluc fragment in NLuc-YF17D** |
| **YF17D-SEQ1** | **CCACCACTCTCTTGACTTTC** |  |

***The template for this reaction is a pre-generated 2A sequence-containing YF17D cDNA clone.**
